# 

**Published:** 1835-07-01

**Authors:** 


### THE MEDICO-CHIRURGICAL REVIEW, AND JOURNAL OF PRACTICAL MEDICINE.

*(NEW SERIES.)*

VOLUME TWENTY-THREE,

[1st of APRIL to 30th of SEPTEMBER,]

1835.

VOL. III. of DECENNIAL SERIES.

EDITED

By JAMES JOHNSON, M. D.

PHYSICIAN EXTRAORDINARY TO THE KING,

AND

HENRY JAMES JOHNSON, Esq.

LECTURER ON ANATOMY AT THE SCHOOL IN KINNERTON STREET, AND FORMERLY HOUSE SURGEON TO ST. GEORGE'S AND THE LOCK HOSPITALS.

Surgeon General's Office LIBRARY 57463 Wash. D.C.

LONDON:

PUBLISHED BY S. HIGHLEY, 32, FLEET STREET,

*Re-printed in New York, by Mr. Wood.*

1835.

W1
ME75T

PRINTED BY G. HAYDEN,
Little College Street, Westminster.

### BIBLIOGRAPHICAL RECORD;

OR,

*Works received for Review since last Quarter.*

1. Consumption, why so fatal? &c. By John Tyrrell. Octavo, pp. 112. Renshaw, April, 1835.

---

2. Principles of the Treatment of Gout, with a further Examination of the Effects of Colchicum, &c. By Sir Charles Scudamore, M.D. F.R.S. &c. Octavo, pp. 54. Longman and Co. 1835.

---

3. The Constitution of Man, considered in relation to external Objects. By Geo. Combe. Second Edition, corrected and enlarged, April, 1835.

---

4. Chemical Attraction. An Essay in

Five Chapters, with an Historical Introduction, and several Illustrative Tables. By GILBERT LANGDON HUME, of Corpus Christi College, Cambridge. Octavo, pp. 176. Cambridge, 1835.

---

5. A few practical Observations on the Art of Cupping. By JOSEPH STAPLES, Cupper, &c. Duodecimo, pp. 72. Longman and Co. April, 1835.

☞ *Concise and practical, the author, according to his motto (ne sutor ultra crepidam), not stepping beyond his just boundary.*

---

6. A Manual of Experiments, illustrative of Chemical Science, &c. By JOHN MURRAY, F.S.A. F.L.S. &c. Fourth Edition, 12mo. Renshaw, 1834.

---

7. A Therapeutic Arrangement and Syllabus of Materia Medica. By JAMES JOHNSTONE, M.D. Fellow of the College of Physicians, and Physician to the General Hospital, Birmingham. Duodecimo, pp. 84. Renshaw, London, 1835.

☞ *Although intended more especially for the Birmingham students, this little manual appears calculated to facilitate the study of materia medica and therapeutics every where.*

---

8. A Dictionary of Practical Medicine, &c. By JAMES COPLAND, M.D. Part III. Octavo, pp. 641 to 690. April, 1835.

☞ *This Part contains* DROPSY *and* FEVER, *inclusive; and maintains its reputation for great research, indefatigable labour, and sound judgment.*

---

9. The Clinique Medicale; or Reports of Medical Cases. By G. ANDRAL: condensed and translated, with Observations extracted from the Writings of the most distinguished Authors. By D. SPILLAN, M.D. &c. Part I. Diseases of the Encephalon. Octavo, pp. 216, very small type, price 5s. Renshaw, May, 1835.

☞ *The value of the original will be appreciated by our readers, from the reviews we have given of it. The translation is admirably condensed, and enriched by valuable notes.*

---

10. A Series of Twenty-eight Plates, illustrating the Causes of Displacement in the various Fractures of the Extremities. By G. W. HIND, M.R.C.S. formerly House-Surgeon to the Middlesex Hospital. Quarto, 1835.

11. The Transactions of the Provincial Medical and Surgical Association, instituted 1832. Volume the third, 8vo, pp. 472. with a Plate. Sherwood and Co. June, 1835.

☞ *To be noticed fully in our next Number.*

---

12. Phrenology, in connexion with the Study of Physiognomy. By S. G. SPURZHEIM, M.D. Illustration of Characters, with 35 Plates. Second American Edition, improved; to which is prefixed a Biography of the Author, by NAHUM CAPEN. Octavo, pp. 367. Boston, 1834.

☞ *This is a very valuable work, especially as relates to the biography of that great and good man*—SPURZHEIM.

---

13. Observations on Mental Derangement, being an Application of the Principles of Phrenology to the Elucidation of the Causes, Symptoms, Nature, and Treatment of Insanity. By ANDREW COMBE, M.D. First American Edition, with Notes and Bibliography, by an American Physician. Octavo, pp. 336. Boston, 1834.

☞ *Dr. Coombe will be gratified to find his work reprinted and improved in the Land of Columbus.*

---

14. Thoughts on Quarantine, and other Sanitary Systems—an Essay which received the Prize of the Boylston Medical Committee of Harvard University, in August, 1834. By CHARLES CALDWELL, M.D. Octavo, pp. 72. Boston, 1834.

☞ *Dr. C. has here given the quarantine system a severe blow; and, as that system is tottering, and must ultimately fall, the author of this brochure has, unquestionably, driven a nail into the coffin of the antique monster.*

---

15. Observations on the Heart, and on the Peculiarities of the Fœtus. By JAMES JEFFRAY, M.D. Professor of Anatomy in the University of Glasgow. Octavo, pp. 112, seventeen Plates. Glasgow, 1835.

---

16. Observations on Atmospheric Influence, chiefly in reference to the Climate and Diseases of Eastern Regions. In five Parts—Part I. By W. AINSLIE, M.D.

☞ *This able Paper is published in a highly useful and well-conducted journal, "the* JOURNAL OF THE ROYAL ASIATIC

Society"—*a work which we strongly recommend to our readers—at least, to all those who are interested in oriental matters.*

---

17. The Cyclopædia of Anatomy and Physiology. Edited by Robert B. Todd, M.B. Lecturer on Anatomy and Physiology at the Westminster School of Medicine, &c. Part the First (to be continued every second Month). Royal 8vo, pp. 112, with numerous Wood-cuts and Engravings, price five shillings. Sherwood and Co. June, 1835.

☞ *This Part contains* Abdomen *(Dr. Todd)*—Absorption *(Dr. Bostock)*—Acalephæ *(Dr. Coldstream)*—Acids, Animal *(W. T. Brande)*—Acrita *(R. Owen)*—Adhesion *(B. Phillips)*—Adipocire *(W. T. Brande)*—Adipose Tissue *(Dr. Craigie)*—Age (*Dr. Symonds*)—Albino (*Dr. Bostock*)—Albumen (*W. T. Brande*)—Amphibia (*T. Bell*)—Animal Kingdom (*Dr. Grant.*)

*As we did not receive this fasciculus till the 4th of June, we could not take particular notice of it in the present Number of the Journal. We read with great attention the article* Age (*with which we have had a longer acquaintance than most* old women, *like ourselves, are prone to acknowledge; and we can vouch for one good article in the present Part, at least. We have glanced at some of the others; and we have no hesitation in prognosticating a most successful issue to the present undertaking, if continued in the same spirit with which it has commenced. When the Cyclopædia of Surgery* (*commencing under the auspices of Mr. Costello*) *shall have been added to the list, we augur a successful competition with our Continental neighbours, in this department of popular medical science.*

---

18. On the Medical Properties of the Natural Order of Ranunculaceæ, more especially on the Uses of the Sabadilla, &c. By A. Turnbull, M.D. Octavo, pp. 171. 1835.

---

19. An Essay on Artificial Teeth, Obturators, and Plates, with Principles for their Construction and Application, illustrated by twenty-six Cases—twenty-one Plates. By Leonard Koecker, Surgeon-Dentist, Doctor in Medicine and Surgery, &c. Octavo, pp. 194, 1835. Highley, Fleet-street.

☞ *We know Mr. Koecker to be a very scientific and expert dentist—and, therefore, we have no doubt but that his observations, in this volume, will prove very serviceable to those who devote their studies to the dental part of surgery. " The object he has in view is twofold:—First, an endeavour to facilitate the labours of his professional brethren—and, secondly, to give that information to the general reader, which will guard him from becoming the dupe of deception and imposture." As Mr. Koecker excels in the dexterity with which he combines science and mechanics, we have no doubt that his strictures on the charlatanism of the lower order of dentists are just, though severe.*

---

20. A Paper on Renal Dropsy, illustrated by Cases and Dissections; read before the Senior Physical Society of Guy's Hospital on the 7th February, 1835. By John Anderson (late Clinical Clerk, Guy's). Octavo, pp. 14, 1835.

☞ *This paper, which has already appeared in the Medical Gazette, is very creditable to Mr. Anderson. We shall make some extracts from it in our next Number.*

---

21. A Formulary for the Preparations and Medicinal Administration of certain new Remedies. Translated from the French of M. Magendie, with Annotations and additional Articles, by Jas. Manby Gully, M.D. Small 8vo, pp. 216. Churchill, London, June, 1835.

---

22. Obstetric Tables; comprising Graphic Illustrations, with Descriptions and Practical Remarks, exhibiting, on dissected Plates, many important Subjects in Midwifery. By G. Spratt, Surgeon-Accoucheur. Second Edition, considerably enlarged and improved, in two Parts—Part the First, quarto, with six dissected Plates. Churchill, June, 1835.

☞ *As the work is dedicated, by permission, to Sir C. M. Clarke, who examined the Plates, and " suggested many improvements," we have the very best authority for their merits and utility.*

---

23. A Theoretical and Practical Treatise on the Diseases of the Skin. By P. Rayer, M.D. Physician to the Hôpital de la Charité, &c. The second Edition, entirely remodelled, with an Atlas, in royal 4to, of 26 Copper Plates, containing four hundred Figures, coloured. One large volume of Letter-press, pp. 1238. Baillière, June, 1835.

☞ *This is an incomparable work on cutaneous diseases. It will be reviewed in our next. Some notes are added by the translator, Dr. Willis.*

